# ^31^P magnetic resonance spectroscopy as a predictor of efficacy in photodynamic therapy using differently charged zinc phthalocyanines

**DOI:** 10.1038/sj.bjc.6690738

**Published:** 1999-10

**Authors:** J C M Bremner, S R Wood, J K Bradley, J Griffiths, G E Adams, S B Brown

**Affiliations:** MRC Radiobiology Unit, Chilton, Didcot, OX11 0RD, UK; School of Biochemistry and Molecular Biology, Department of Colour Chemistry and Dyeing, Centre for Photobiology and Photodynamic Therapy, University of Leeds, Leeds, LS2 9JT, UK

**Keywords:** photodynamic therapy, magnetic resonance, phthalocyanines

## Abstract

Photodynamic therapy (PDT) is a developing approach to the treatment of solid tumours which requires the combined action of light and a photosensitizing drug in the presence of adequate levels of molecular oxygen. We have developed a novel series of photosensitizers based on zinc phthalocyanine which are water-soluble and contain neutral (TDEPC), positive (PPC) and negative (TCPC) side-chains. The PDT effects of these sensitizers have been studied in a mouse model bearing the RIF-1 murine fibrosarcoma line studying tumour regrowth delay, phosphate metabolism by magnetic resonance spectroscopy (MRS) and blood flow, using D_2_O uptake and MRS. The two main aims of the study were to determine if MRS measurements made at the time of PDT treatment could potentially be predictive of ultimate PDT efficacy and to assess the effects of sensitizer charge on PDT in this model. It was clearly demonstrated that there is a relationship between MRS measurements during and immediately following PDT and the ultimate effect on the tumour. For all three drugs, tumour regrowth delay was greater with a 1-h time interval between drug and light administration than with a 24-h interval. In both cases, the order of tumour regrowth delay was PPC > TDEPC = TCPC (though the data at 24 h were not statistically significant). Correspondingly, there were greater effects on phosphate metabolism (measured at the time of PDT or soon after) for the 1-h than for the 24-h time interval. Again effects were greatest with the cationic PPC, with the sequence being PPC > TDEPC > TCPC. A parallel sequence was observed for the blood flow effects, demonstrating that reduction in blood flow is an important factor in PDT with these sensitizers. © 1999 Cancer Research Campaign


*British Journal of Cancer* (1999) **81**(4), 616–621
© 1999 Cancer Research Campaign
Article no. bjoc.1999.0738

### $^{31}P$ magnetic resonance spectroscopy as a predictor of efficacy in photodynamic therapy using differently charged zinc phthalocyanines

**JCM Bremner[1], SR Wood[2,4], JK Bradley[1], J Griffiths[3,4], GE Adams[1] and SB Brown[2,4]**

[1]MRC Radiobiology Unit, Chilton, Didcot OX11 0RD, UK; [2]School of Biochemistry and Molecular Biology, [3]Department of Colour Chemistry and Dyeing and [4]Centre for Photobiology and Photodynamic Therapy, University of Leeds, Leeds LS2 9JT, UK

**Summary** Photodynamic therapy (PDT) is a developing approach to the treatment of solid tumours which requires the combined action of light and a photosensitizing drug in the presence of adequate levels of molecular oxygen. We have developed a novel series of photosensitizers based on zinc phthalocyanine which are water-soluble and contain neutral (TDEPC), positive (PPC) and negative (TCPC) side-chains. The PDT effects of these sensitizers have been studied in a mouse model bearing the RIF-1 murine fibrosarcoma line studying tumour regrowth delay, phosphate metabolism by magnetic resonance spectroscopy (MRS) and blood flow, using $D_2O$ uptake and MRS. The two main aims of the study were to determine if MRS measurements made at the time of PDT treatment could potentially be predictive of ultimate PDT efficacy and to assess the effects of sensitizer charge on PDT in this model. It was clearly demonstrated that there is a relationship between MRS measurements during and immediately following PDT and the ultimate effect on the tumour. For all three drugs, tumour regrowth delay was greater with a 1-h time interval between drug and light administration than with a 24-h interval. In both cases, the order of tumour regrowth delay was PPC > TDEPC = TCPC (though the data at 24 h were not statistically significant). Correspondingly, there were greater effects on phosphate metabolism (measured at the time of PDT or soon after) for the 1-h than for the 24-h time interval. Again effects were greatest with the cationic PPC, with the sequence being PPC > TDEPC > TCPC. A parallel sequence was observed for the blood flow effects, demonstrating that reduction in blood flow is an important factor in PDT with these sensitizers. © 1999 Cancer Research Campaign

**Keywords:** photodynamic therapy; magnetic resonance; phthalocyanines

Photodynamic therapy (PDT) with Photofrin has now been approved by regulatory authorities in several countries and an increasing number of patients are being treated. Nevertheless, the need for new clinical photosensitizers with improved purity, tumour selectivity, light absorption profile, photophysical properties and tissue clearance has been recognized (Bellnier and Henderson, 1992; Dougherty, 1993). Phthalocyanines have the potential to fulfil many of these criteria (Ben Hur, 1992; Fingar et al, 1993; Griffiths et al, 1994) but, although many potential candidates have been studied experimentally (Spikes, 1986; Ben Hur et al, 1987), there has been little clinical application and that appears to have been restricted largely to the use of aluminium sulphonated phthalocyanine in Russia (Stranadko et al, 1996).

We have developed a series of novel photosensitizers based on zinc(II) phthalocyanine, rendered water-soluble by appropriate substitution at the periphery of the molecule (Cruse-Sawyer et al, 1998). Three such sensitizers are of particular interest in mechanistic terms, since they contain negatively charged, positively charged and neutral peripheral groups respectively (Figure 1). The substitution does not significantly alter the photophysical properties of the molecules in terms of absorption spectra and singlet oxygen generation and the compounds therefore offer a means of comparatively assessing the effects of charge on such parameters as uptake into tumours and other tissues, pharmacokinetics, sub-tumoural and sub-cellular distribution and PDT efficacy.

One of the complicating features of PDT is the large number of treatment parameters which have to be set compared with other modalities. These include choice of drug, drug dose, light dose, light dose rate, drug-time interval and mode of light delivery (e.g. interstitial versus surface illumination). Optimization of these parameters for particular indications is difficult and is associated with variability in outcome. It would be extremely valuable if a physical technique could be used to predict PDT efficacy at the time of treatment, when appropriate adjustment to at least some of these treatment parameters would be possible. In principle, magnetic resonance spectroscopy (MRS) offers this possibility through measurement of phosphate metabolism during and immediately after light irradiation. Work in several laboratories has shown that $^{31}P$ MRS does indeed reveal early changes in phosphate metabolism either during or following PDT (Ceckler et al, 1986; Chopp et al, 1987; Hilf et al, 1987; Mattiello et al, 1990; Chapman et al, 1991; Bremner et al, 1994).

The main aims of the current paper are twofold. First, we have carried out a MRS study in a mouse model, using the three novel cationic, anionic and neutral zinc phthalocyanines developed at Leeds, to determine if measurements made at the time of PDT treatment can be predictive of ultimate PDT efficacy. Secondly, we

*Received 26 November 1998*
*Revised 22 April 1999*
*Accepted 26 April 1999*

*Correspondence to:* SB Brown; School of Biochemistry and Molecular Biology, University of Leeds, Leeds LS2 9JT, UK

This paper was prepared with the full participation of Professor GE Adams, before his death on 6 June 1998.

**Table 1** Regrowth delay (± s.e.m.) induced in RIF-1 tumours by the different PCs following a 50 J cm$^{-2}$ dose of light when the drug–light time interval (TL) was either 1 or 24 h

| PC (10 mg kg$^{-1}$) | Regrowth delay (days) TL = 1 h | TL = 24 h |
|---|---|---|
| PPC | 12.5 ± 1.8 | 6.1 ± 1.1 |
| TCPC | 8.2 ± 1.7 | 4.6 ± 1.4 |
| TDEPc | 7.8 ± 0.9 | 3.0 ± 1.3 |

All mice were anaesthetized and six animals were used per group. The tumour growth delays were calculated as described in Materials and Methods.

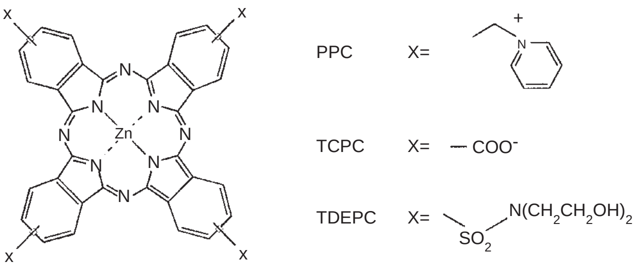

**Figure 1** The chemical structure of the phthalocyanines PPC, TCPC and TDEPC

have used the same three sensitizers to assess the effects of charge on PDT efficacy and whether any MRS correlation is itself dependent on the sensitizer charge. As a secondary aim, we have taken advantage of the ability of MRS to measure blood flow immediately following PDT under the same conditions used in the main study, to assess whether reduction in blood flow represents an important mechanism of tumour damage using these sensitizers.

### MATERIALS AND METHODS

### Photosensitizers

Three phthalocyanines differing in the structure of their constituent side-chains and overall net charge (Figure 1) were synthesized in the Department of Colour Chemistry, University of Leeds. Tetradihydroxyethylsulphonamide zinc phthalocyanine (TDEPC), Pyridinium methyl zinc phthalocyanine (PPC) and tetracarboxy zinc phthalocyanine (TCPC) have neutral, positive and negative charges respectively and all are water-soluble. The preparation and characterization of these compounds have been described previously (Griffiths et al, 1997).

Each compound was dissolved in isotonic saline to a concentration of 2 mg ml$^{-1}$ and administered intravenously (i.v.) at a dose of 10 mg kg$^{-1}$.

### Tumour model

The RIF-1 murine sarcoma line was maintained as described previously (Twentyman et al, 1980; Stratford et al, 1988). Approximately $2 \times 10^5$ cells suspended in 0.05 ml phosphate-buffered saline (PBS) were implanted intradermally (i.d.) into the mid-dorsal pelvic region of 8- to 10-week-old male C3H/He mice (category IV).

### Tumour regrowth delay

The tumours were measured in three orthogonal directions (a, b and c) using graduated vernier callipers. Their volumes (V) were calculated using the formula $V = abc \times \pi/6$. The mice were used for treatment when their tumours had reached a volume of 80–160 mm$^3$. Following treatment, the tumours were measured three times per week and the time taken for each tumour within a group to reach four times its volume at the start of treatment was calculated. Six animals per group were used and the mean (± s.e.m.) of the regrowth times for each treated group was calculated. From these data the growth delay was calculated as Texp–Tcon, where Tcon and Texp are the mean times taken to reach four times volume for the control and relevant experimental groups respectively.

### Anaesthetic

For both the growth delay and MRS studies the mice were anaesthetized using a 1:1:2 mixture of Hypnorm/Hypnovel/water 20 min prior to giving the light dose.

### Light source

A Spectra Physics 2016-6W argon ion-pumped dye laser was used to generate light at a given wavelength by tuning the birefringent filter. The light was then directed down a fibreoptic cable which ran directly from the laser facility to the magnetic resonance building. A 200-μm diameter core hard clad silica (HCS) single fibre with a ruggedized external sheathing was used. This was coupled to the laser light using a multimode fibre coupler (Newport Corporation). The end of this fibre (230 μm outer diameter) was cleaved and inserted interstitially into the centre of the tumour, parallel to the body of the mouse. Only one fibre was required for each tumour.

The power density of the light, measured before insertion, was 100 mW cm$^{-2}$ and the duration of the exposure was 500 s to give a total light dose of 50 J cm$^{-2}$. The wavelengths of light used to activate TDEPC, PPC and TCPC were 680, 680 and 692 nm respectively (Griffiths et al, 1994) (Figure 2). The time interval between giving the photosensitizer and the light dose was either 1 or 24 h.

### Magnetic resonance spectroscopy

All experiments were performed in a 4.7 Tesla superconducting magnet with a 30 cm horizontal bore (Oxford Instruments) connected to a SISCO 200 spectrometer (Varian Associates) (Bremner et al, 1994). The body temperature of the anaesthetized mice in the magnet was maintained at 34–35°C by a device for circulating hot water.

#### *Phosphorus metabolism*

A two-turn, 7-mm diameter surface coil that fitted neatly over the tumour was used both as transmitter and receiver. Each spectrum accumulated consisted of 256 acquisitions collected in 512 s, using a 2 s repetition rate, 0.1 s acquisition time and a spectral width of 5000 Hz.

Prior to placement in the magnet, the optical fibre was inserted into the tumour of the anaesthetized mouse, and the mouse was

© 1999 Cancer Research Campaign then positioned in the centre of the magnet. For each mouse a control spectrum was collected and then, without moving the mouse, the light irradiation was given (Bremner et al, 1994). In this way it was possible to store the spectrum once halfway through the irradiation time (i.e. after 128 scans – 4 min) and again at the end (after 256 scans). Spectra were then collected immediately after irradiation and subsequently at 10-min intervals for 1 h. At 24 h following treatment the unanaesthetized mice were lightly restrained in plastic jigs and replaced in the centre of the magnet where a final spectrum was obtained.

The spectra were analysed as described previously (Bremner et al, 1994) and the ratio of the area under the inorganic phosphate (Pi) peak to the total area under all the peaks (Pi/total) was used to indicate the changes occurring in the spectra and hence in phosphate metabolism. As cell mitochondrial function is damaged (due to PDT, for example) the Pi/total P ratio increases and hence this ratio can be used as an early measure of cell damage. As we have previously reported (Bremner et al, 1991) when the Pi/total ratio is above approximately 0.4 it is difficult to distinguish the level of ATP from the background noise. Therefore any increase in the ratio above this value is likely to be due to a change in the Pi:phosphomonoester ratio and not to further changes in ATP levels. The time points on the graphs correspond to the mid-points of the collection times.

For statistical analysis, a Student's *t*-test ($P < 0.05$) was used to obtain a comparison of Pi/total ratios for the treated and control groups.

### *Deuterium ($D_2O$) uptake measurements*

A five-turn solenoid surface coil of 1-cm diameter was used (Bradley et al, 1994). Each spectrum was acquired in 15 s and contained averaged data from 75 scans at a repetition time of 0.2 s, 0.1-s acquisition time and a spectral width of 2500 Hz. For each uptake measurement an array of 70 spectra were obtained in 20 min. Relative HDO concentrations were estimated from the height of the HDO peaks.

A cannula (Jelco, 24 gauge) rinsed with heparin and connected to silicone tubing was inserted into the tail vein of the mouse. The tubing was filled with $D_2O$–saline (0.9% w/v sodium chloride) and connected to a 1-ml syringe situated outside the magnet bore. In the mouse, $D_2O$ becomes a tracer molecule (HDO) through the rapid exchange of protons. In order to obtain a background level of HDO, five complete spectra were obtained before injecting 70 µl of $D_2O$ during the 6th scan.

A control HDO uptake rate was measured immediately before light irradiation for each anaesthetized mouse and then further measurements were made 5, 30, 60 min and 24 h after treatment. The analysis of the $D_2O$ data has been described in detail previously (Bradley et al, 1994; Bremner et al, 1994). The tumour blood flow (TBF) following treatment is calculated as a value relative to its own control. The time points on the graphs (Figure 3) correspond to the mid-points of the collection times.

### RESULTS

### Photodynamic therapy

The growth time for the RIF-1 tumours in the control (no drug and no light) animals was 7.7 ± 0.1 days to reach four times original tumour volume. This represents the Tcon parameter defined in the Materials and Methods section. Under the PDT conditions chosen, a significant effect on tumour regrowth for the RIF-1 tumours was found for each of the sensitizers ($P < 0.05$), demonstrating that each sensitizer was effective in tumour control. Values for the regrowth delay, as defined in Materials and Methods, with each of the phthalocyanines, are shown in Table 1. With drug alone or with light alone there was no significant regrowth delay.

Table 1 clearly shows that when the drug–light interval was 1 h, regrowth delay was greater than when the interval was 24 h for each of the sensitizers, presumably due to clearance of the drug from the tumours. It is also apparent that there are differences in efficacy between the sensitizers. At the 1-h drug–light time interval, the order of effectiveness was PPC > TDEPC = TCPC. Analysis of variance (ANOVA) demonstrated that PPC was significantly different to TDEPC ($P < 0.05$) but there was no significant difference between TDEPC and TCPC. At the 24-h time interval,

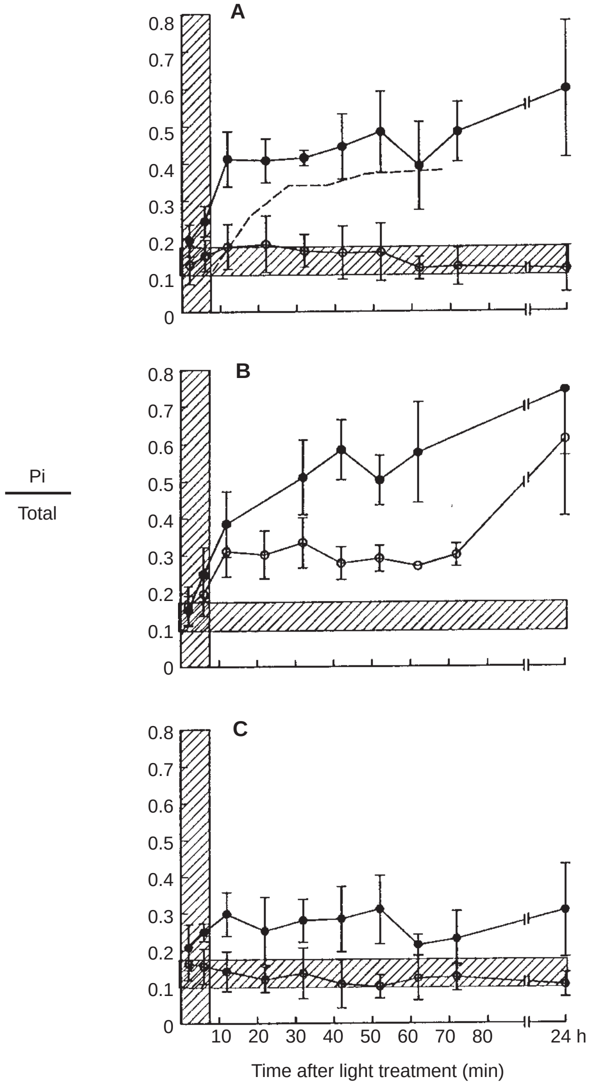

**Figure 2** PDT-induced changes in the Pi/total ratio during and after a light dose of 50 J cm$^{-2}$ with either TDEPC (**A**), PPC (**B**) or TCPC (**C**). The drugs were given i.v. at a dose of 10 mg kg$^{-1}$. Results are shown for TL values of 1 (●) or 24 h (○). The vertical hatched line indicates the duration of light treatment (500 s). The horizontal hatched lines shows the Pi/total ratios (mean ± s.e.m.) observed in untreated control tumours. The dotted line in **A** shows the changes in the ratio occurring when tumour blood flow is occluded using mechanical clamps

*© 1999 Cancer Research Campaign*

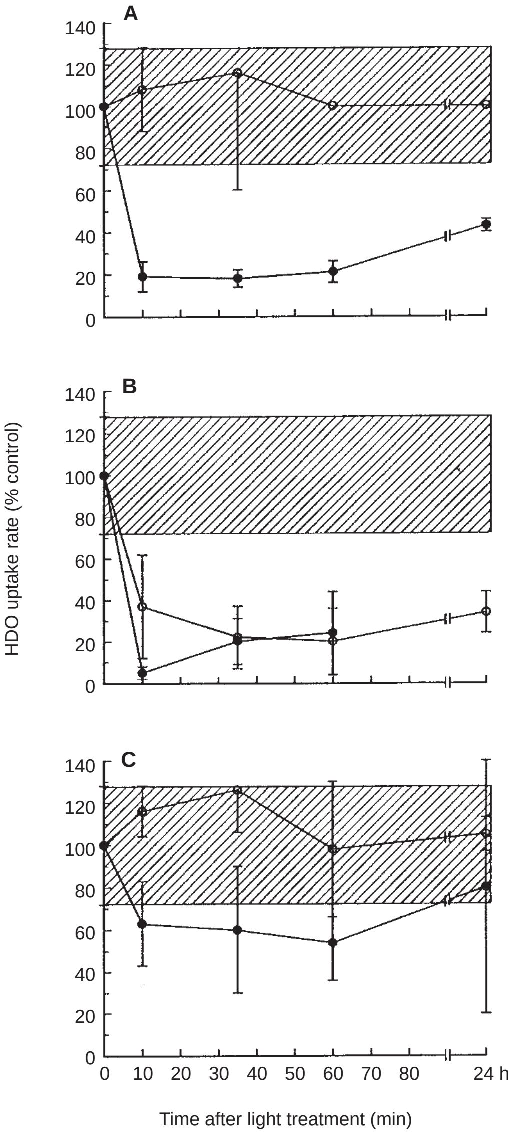

**Figure 3** The relative changes in blood flow as measured by deuterium uptake against time after a PDT light dose of 50 J cm$^{-2}$ with either TDEPC (**A**), PPC (**B**) or TCPC (**C**). Results are shown for TL values of 1 (●) or 24 h (○). The change in uptake rate of HDO is plotted as a percentage of the control value again the order of effectiveness appeared to be PPC > TDEPC = TCPC, though ANOVA demonstrated no significant differences at this time point.

It should be emphasized that, in this study, the aim was not to use conditions which produced maximum PDT effects, but to use conditions which produced different PDT effects, both for the different sensitizers and for the different drug–light intervals, so that the variation in efficacy could be used to assess correlation with the MRS data. In this respect, therefore, the conditions have clearly proved appropriate.

### $^{31}$P magnetic resonance spectroscopy

Figure 2 shows the changes induced in Pi/total ratios obtained from the phosphorus spectra following a PDT dose of 50 J cm$^{-2}$ when the three PCs were administered either 1 or 24 h before the light. It is clear that, for each sensitizer at the 1-h drug–light time interval, there were substantial initial effects on the phosphorus spectrum, in agreement with previous studies with other sensitizers. This result shows for the first time that, with these sensitizers, there are rapid changes in phosphorus metabolism during or immediately after PDT and that these changes may be assessed in relation to their use in predicting ultimate PDT outcome. However, when the drug–light time interval was 24 h, the effects were much less. They were smaller for PPC but, for TDEPC and TCPC, they were not distinguishable from controls.

For the 1-h drug–light time interval, a more detailed analysis shows that, over the first hour following PDT, the effect is in the sequence PPC > TDEPC > TCPC, though distinction between PPC and TDEPC at short times would be difficult. For the 24-h drug–light time interval, the corresponding order is PPC > TDEPC = TCPC. Under all conditions where an effect relative to control was observed, the Pi/total ratio increased rapidly initially, then plateaued after 10–30 min up until at least 60–70 min, except in the case of TCPC where the already small effect decreased further after about 50 min. It was not possible to carry out a series of longer time measurements, but it was possible to carry out a single measurement at 24 h in each case. Interestingly, without exception, these values show a significant increase over the plateau level.

For one sensitizer, TDEPC, the effect on the Pi/total ratio of occlusion of the blood supply by a metal clamp was studied. Results are shown by the dotted line in Figure 2A for a 1-h drug–light interval. Although this study used only one animal per time point, the trend suggests that the initial effects of PDT on phosphorus metabolism appear to be substantially smaller with a clamped vessel.

### Blood flow

Figure 3 shows measurements of blood flow in terms of the relative HDO uptake rate for all three sensitizers, as described in Materials and Methods. When the drug–light interval was 1 h, PDT treatment with both TDEPC and PPC (Figure 3 A, B) reduced the tumour blood flow by 80 and 95% of control values respectively, within the first 10 min post-treatment. The PDT effect on blood flow with TCPC (Figure 3C) showed only about a 40% decrease, i.e. the sequence was PPC > TDEPC > TCPC. In all three cases, the initial reduction in blood flow was maintained for at least the first hour and rose only slightly after 24 h.

When the drug–light interval was 24 h, the tumour blood flow remained within the range expected for untreated tumours for up to 24 h following light treatment with either TDEPC or TCPC. However, with PPC, the tumour blood flow fell by approximately 60% of the control level within 10 min post-treatment and this reduction was maintained for up to 24 h, i.e. the sequence was PPC > TDEPC = TCPC.

### DISCUSSION

### Correlation between PDT efficacy and MRS measurements

One of the two main objectives addressed by the present paper is the question of whether differences in the PDT efficacy, measured many days after treatment and representing both variation in sensitizer and

© 1999 Cancer Research Campaign variation in drug–light interval, might be reflected in the MRS measurements taken during and immediately after PDT. In broad terms, it is clear that there is indeed such correlation. Considering variation in sensitizer, at the 1-h interval, PPC was the most effective in PDT and also gave the greatest effect on phosphate metabolism. Comparing PPC itself at the two time intervals also shows broad correlation between growth delay and initial change in phosphate metabolism. However, these correlations cannot be pressed too far from the present data since, at the 1-h interval, although TDEPC showed an apparently greater phosphate metabolism effect than TCPC, their PDT efficacies were not distinguishable. Moreover, at the longer time interval, there were no significant differences in phosphate metabolism above control values, yet both sensitizers did give a definite PDT effect.

The present data suggest, therefore, that MRS measurements certainly have the potential to be predictive of ultimate PDT effect and, even in this early work, the ability to reflect gross differences in PDT efficacy has been demonstrated. However, the correlation is not precise and certainly far from quantitative at present.

### Effects of charge on PDT efficacy

Since there is little difference in absorption or photophysical properties between the three sensitizers (Griffiths et al, 1997), any differences in PDT effectiveness are likely to be attributable to differences in uptake and hence sub-tumoural and sub-cellular distribution. Charge on the sensitizer is likely to play a major role in this though it is, of course, not possible to carry out a true control (i.e. same chemical substituent but different charge). PPC was clearly the most effective sensitizer used in terms of growth delay at both time intervals and it seems likely that this was due to its positive charge. Interestingly, however, there was little difference between the neutral and the negatively charged phthalocyanine. To date there have been few studies of neutral photosensitizers, in part because of problems with water-solubility. Similar results were found for comparison of the same or similar sensitizers in a transplanted fibrosarcoma in the rat (Cruse-Sawyer et al, 1998). Both this study and the recent study referred to above (Cruse-Sawyer et al, 1998) have suggested that the cationic phthalocyanine is the most effective in PDT. It was not possible to determine octanol:water partition coefficients because, for each sensitizer, almost all of the sensitizer was found in the aqueous layer. The effects seen here therefore do not appear to be strongly related to hydrophobicity/hydrophilicity considerations. It therefore seems likely that the cationic sensitizer is most effective, primarily because of its positive charge, although as stated earlier, it is not possible to do a real control and studies with other cationic sensitizers would be necessary to investigate this effect further.

### Blood flow measurements

All three sensitizers showed a marked and rapid reduction in blood flow for the 1-h time interval, as did PPC for the 24-h interval. This demonstrates that early vascular shutdown occurs during PDT with all three sensitizers. Moreover, the reduction in blood flow almost exactly parallels the behaviour of the Pi/total ratio. The broad correlation drawn between PDT efficacy and the phosphate metabolism data therefore applies equally to the relationship between PDT efficacy and blood flow. The clamping experiments also appear to demonstrate the importance of vasculature effects to PDT with this sensitizer, though such experiments are preliminary and further work is needed for a thorough interpretation of such data. It is therefore reasonable to assume that vascular shutdown and consequent hypoxia represents a major part of the mechanism of growth delay for these sensitizers with this tumour.

### General discussion

The present work has clearly demonstrated that there is a relationship between MRS measurements during and immediately following PDT and the ultimate effect on the tumour and that, potentially, these measurements may be used to predict that ultimate effect. Clearly, much more work would be necessary before such predictions could be quantitative. The main clinical value of such correlation would be the ability it would impart to adjust treatment parameters at the time of treatment to ensure a successful outcome, by increasing the PDT effect if the MRS predictor suggested that treatment would be incomplete. Any adjustment would be limited to factors which could be manipulated at treatment time (clearly, drug identity and drug dose would already have been fixed). Such factors could include total light dose, light dose rate, fractionation of the light dose and possibly oxygen concentration. Further study in animal tumours using the sensitizers employed in this work or other sensitizers will be necessary to evaluate the effects of these parameters.

### ACKNOWLEDGEMENTS

We wish to thank Carol Williams, Kevin Bennett and Debbie Pocock for their help with the experiments and also Ian Stratford for his helpful discussion of the results. SRW and SBB would also like to thank Yorkshire Cancer Research for their generous financial support.

### REFERENCES

Bellnier DA and Henderson BW (1992) Determinants for photodynamic tissue destruction. In: *Photodynamic Therapy: Principles and Clinical Applications*, Henderson BW and Dougherty TJ (eds), pp. 117–127. Marcel Dekker: New York

Ben-Hur E (1992) Basic photobiology and mechanisms of action of phthalocyanines. In: *Photodynamic Therapy: Basic Principles and Clinical Applications*, Henderson BW and Dougherty TJ (eds), pp. 63–78. Marcel Dekker: New York

Ben-Hur E, Green M, Prager A, Kol R and Rosenthal I (1987) Phthalocyanine photosensitization of mammalian cells: biochemical and ultrastructure effects. *Photochem Photobiol* **46**: 651–656

Bradley JK, Counsell CJR, Bremner JCM, Sansom JM and Adams GE (1994) The accuracy and reproducibility of measuring blood flow in murine tumours by the $D_2O$ uptake and clearance techniques. *NMR Biomed* **7**: 141–148

Bremner JCM, Counsell CJR, Adams GE, Stratford IJ, Wood PJ, Dunn JF and Radda GK (1991) In vivo 31P muclear magnetic resonance spectroscopy of experimental murine tumours and human tumour xenografts: effects of blood flow modification. *Br J Cancer* **64**: 862–866

Bremner JCM, Bradley JK, Stratford IJ and Adams GE (1994) Magnetic resonance spectroscopic studies on ‘real-time’ changes in the RIF-1 tumour metabolism and blood flow during and after photodynamic therapy. *Br J Cancer* **69**: 1083–1087

Ceckler TL, Bryant RG, Penney DP, Gibson SL and Hilf R (1986) $^{31}P$ NMR spectroscopy demonstrates decreased ATP levels in vivo as an early response to PDT. *Biochem Biophys Res Commun* **140**: 273–279

Chapman JD, McPhee MS, Walz N, Chetner MP, Stobbe CC, Soderlind K, Arnfield M, Meeker BE, Trimble L and Allen PS (1991) Nuclear magnetic resonance spectroscopy and sensitizer-adduct measurements of photodynamic therapy-induced ischemia in solid tumors. *J Natl Cancer Inst* **83**: 1651–1659

Chopp M, Farmer H, Hetzel FW and Schaap AP (1987) In vivo $^{31}P$ NMR spectroscopy of mammary carcinoma subjected to subcurative photodynamic therapy. *Photochem Photobiol* **45**: 819–821

 © 1999 Cancer Research Campaign

Cruse-Sawyer JE, Griffiths J, Dixon B and Brown SB (1998) The photodynamic response of two rodent tumour models to four zinc (II)-substituted phthalocyanines. *Br J Cancer* **77**: 965–972
Dougherty TJ (1993) Photodynamic therapy. *Photochem Photobiol* **58**: 895–900
Fingar VH, Wieman TJ, Karavolos PS, Doak KW, Ouellet R and van Lier JE (1993) The effects of PDT using differently substituted zinc phthalocyanines on vessel constriction, leakage and tumour response. *Photochem Photobiol* **58**: 251–258
Fingar VH, Wieman TJ, Karavolos PS, Doak KW, Ouellet R and van Lier JE (1993) The effects of PDT using differently substituted zinc phthalocyanines on vessel constriction, leakage and tumour response. *Photochem Photobiol* **58**: 251–258
Griffiths J, Cruse-Sawyer J, Wood SR, Schofield J, Brown SB and Dixon B (1994) On the photodynamic therapy action spectrum of zinc phthalocyanine tetrasulphonic acid in vivo. *J Photochem Photobiol* B **24**: 195–199
Griffiths J, Schofield J, Wainwright M and Brown SB (1997) Some observations on the synthesis of polysubstituted zinc phthalocyanine sensitisers for photodynamic therapy. *Dyes Pigments* **33**: 65–78
Hilf R, Gibson SL, Penny DP, Ceckler TL and Bryant RG (1987) Early biochemical responses to photodynamic therapy monitored by NMR spectroscopy. *Photochem Photobiol* **46**: 809–817
Mattiello J, Eveloch JL, Brown E, Schaap AP and Hetzel FW (1990) Effect of photodynamic therapy on RIF-1 tumour metabolism and blood flow examined by $^{31}$P and $^{2}$H NMR spectroscopy. *NMR Biomed* **3**: 64–70
Spikes JD (1986) Phthalocyanines as photosensitizers in biological systems and for the photodynamic therapy of tumours. *Photochem Photobiol* **43**: 691–699
Stranadko EF, Skobelkin OK, Litwin GD and Astrakhankina TA (1996) *Proc SPIE* **2728**: 194–205
Stratford IJ, Adams GE, Godden J and Howells N (1988) Potentiation of the anti-tumour effect of melphalan by the vaso-active drug, hydralazine. *Br J Cancer* **58**: 122–127
Twentyman PR, Brown JM, Gray JW, Franko AJ, Scoles MA and Kaleman RF (1980) A new mouse tumour model system (RIF-1) for comparison of end-point studies. *J Natl Cancer Inst* **64**: 595–604
Wood SR, Holroyd JA and Brown SB (1997) The subcellular localisation of Zn(II) phthalocyanines and their redistribution on exposure to light. *Photochem Photobiol* **65**: 397–402

© 1999 Cancer Research Campaign
